# Swarming bacteria exhibit developmental phase transitions to establish scattered colonies in new regions

**DOI:** 10.1093/ismejo/wrae263

**Published:** 2025-01-03

**Authors:** Amanda M Zdimal, Giacomo Di Dio, Wanxiang Liu, Tanya Aftab, Taryn Collins, Remy Colin, Abhishek Shrivastava

**Affiliations:** Center for Fundamental and Applied Microbiomics, Biodesign Institute, Arizona State University, Tempe, AZ 85287, United States; School of Life Sciences, Arizona State University, Tempe, AZ 85287, United States; Department of Systems and Synthetic Microbiology, Max Planck Institute for Terrestrial Microbiology, Marburg 35043, Germany; Center for Fundamental and Applied Microbiomics, Biodesign Institute, Arizona State University, Tempe, AZ 85287, United States; School of Life Sciences, Arizona State University, Tempe, AZ 85287, United States; Center for Fundamental and Applied Microbiomics, Biodesign Institute, Arizona State University, Tempe, AZ 85287, United States; Center for Fundamental and Applied Microbiomics, Biodesign Institute, Arizona State University, Tempe, AZ 85287, United States; Department of Systems and Synthetic Microbiology, Max Planck Institute for Terrestrial Microbiology, Marburg 35043, Germany; Center for Fundamental and Applied Microbiomics, Biodesign Institute, Arizona State University, Tempe, AZ 85287, United States; School of Life Sciences, Arizona State University, Tempe, AZ 85287, United States

**Keywords:** swarming, collective behavior, bacterial motility, bacterial colonization, biofilms

## Abstract

The collective surface motility and swarming behavior of microbes play a crucial role in the formation of polymicrobial communities, shaping ecosystems as diverse as animal and human microbiota, plant rhizospheres, and various aquatic environments. In the human oral microbiota, T9SS-driven gliding bacteria transport non-motile microbes and bacteriophages as cargo, thereby influencing the spatial organization and structural complexity of these polymicrobial communities. However, the physical rules governing the dispersal of T9SS-driven bacterial swarms are barely understood. Here, we collected time-lapse images, under anaerobic conditions, of developing swarms of a T9SS-driven microbe common to the human oral microbiota. Tracking of swarms revealed that small peripheral flares emerging from a colony develop structures that resemble fireworks displaying a chrysanthemum effect and flower-like patterns that convert to wave-like patterns and which further evolve into scattered microcolonies. Particle-image velocimetry showed density-dependent phase transitions and initial vorticity within these emerging patterns. Numerical simulations demonstrate that these patterns arise due to changes in swarm speed and alignment strength. Our data reveal a strategy used by an anaerobic swarming bacterium to control swarm behavior, resulting in scattered microcolonies distant from the mother colony, thus reducing competition for resources among colony members. This might ensure species survival even if conditions change drastically in one location of the human oral cavity.

## Graphical Abstract

**Graphical Abstract:**
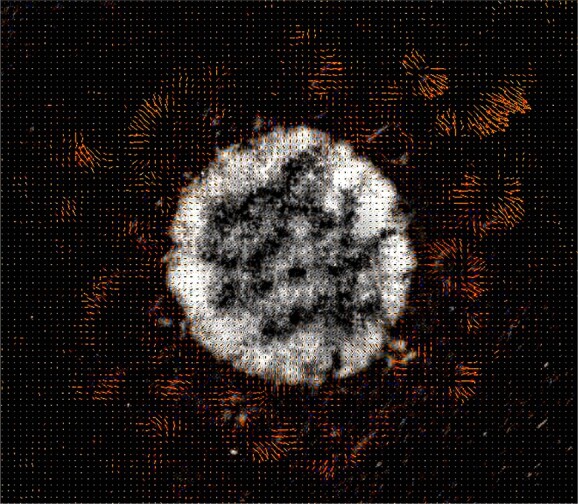

## Introduction

Collective motion is common in biology, on both microscopic and macroscopic levels [1, 2]. The widespread use of coordinated movements amongst members of a group implies evolutionary benefits of these behaviors [3]. Motility is imperative in the search for favorable environmental conditions and scavenging for water and adequate food sources. The ability of many members of a group to mobilize ensures survival of the species from predators, waste accumulation, and environmental challenges. Although motility and collective motion are found to often provide benefits, the mechanisms controlling these behaviors in bacteria are poorly understood.

Motile bacteria serve as excellent models for studying the mechanics of motility and collective motion in their simplest forms. Bacteria have developed various strategies for movement, highlighting the significance of mobility across all scales of life. Most bacteria rely on extracellular appendages to facilitate movement; for instance, type IV pili enable twitching motility [4], and flagella allow bacteria to swim through liquids and swarm across surfaces. The well-characterized flagellar motility is driven by proton motive force (pmf) and it is arguably the most common type of motility utilized by bacteria [5, 6]. Another recently discovered motor that drives motility is the Type 9 secretion system (T9SS), which transports essential proteins across the outer membrane to execute gliding motility [7]. T9SS is one of the three known pmf-driven biological rotary motors, the other two being ATP synthase and the bacterial flagellar motor [8, 9]. In the T9SS system, a rotary motor powered by proton motive force drives the transport of cell surface adhesins across the surface in helical fashion [10, 11]. The adhesins attach to a helical track on the cell surface and interact with an external substratum such that the movement of the adhesin across the cell surface propels the cell forward [12, 13]. Gliding machinery is common across the Bacteroidetes phylum, but not all T9SS-containing organisms are capable of motility.

Bacteria of the genus *Capnocytophaga*, typically isolated from the human oral microbiota, are driven by T9SS and serve as potential models for studying gliding motility and its impact on host-associated polymicrobial communities. *Capnocytophaga* sp. coordinate gliding motility between cells to result in collective motion through a process known as swarming [14–16]. Interestingly, *Capnocytophaga* is the only genus in the human oral cavity that utilizes T9SS-driven gliding motility, marking it as an important representative of the Bacteroidetes phylum in the human oral microbiota [17]. The gliding motility and T9SS of *Capnocytophaga ochracea* is crucial for the formation of single-species biofilms [18]. The genus *Capnoctophaga* is one of the prominent bacterial genera in the oral microbiome, reaching colonization levels up to 8% of the total bacteria present in the supra-gingival and sub-gingival plaques of healthy humans [19]. Studies aimed at characterizing the role of *Capnocytophaga* sp. in the oral cavity are minimal but have shown its ability to swarm and carry other bacteria [20], as well as bacteriophages [21] as hitchhikers. *Capnocytophaga* sp. are detected in plaque deposits tenfold higher than in non-plaque sites in healthy individuals [19]. Additionally, it is also considered as an opportunistic human pathogen [22, 23]. Taken together, *Capnocytophaga sp.* appears to play a complex and crucial role in shaping the structure of the human oral microbiota and overall human health.

One challenge in studying swarm development of *Capnocytophaga* sp. is that it requires anaerobic conditions for growth and motility. Bacteria currently used as models for swarming behavior thrive in aerobic conditions, which allows for easy observation of swarm development and structure [24–29]. Many of these studies have shown that bacteria display unique phenotypes with different swarm sizes and patterns. Swarms of aerobic bacteria have been analyzed for properties that include pattern formation, speed, vorticity, and the direction of movement. Due to the interest in characterizing swarm development, many models have been developed that aim to facilitate our understanding of swarm processes [30–38]. These models demonstrate the phase separation of particles [39] and multiple studies have confirmed the presence of phase transitions in bacterial swarms that align with numerical simulations [40–42]. However, to our knowledge, no study has used an anaerobic swarming bacterium as a model for collective motion.

We investigated motility and swarm development of *C. ochracea* under anaerobic conditions. Surface stiffness and nutrient composition were altered to assess how these factors influence swarm capabilities of *C. ochracea.* We quantified changes in swarm size and patterns as a function of varying agar concentration and nutrient composition. Long-term imaging revealed that *C. ochracea* swarms exhibit striking spatiotemporal patterns reminiscent of fireworks displaying a chrysanthemum effect, characterized by circular forms with jagged edges. Later, one half of the flower-like structure expands outwards, transitioning into wave-like patterns. Timelapses of developing *C. ochracea* swarms were captured, and their movements were tracked using a modified version of classical particle image velocimetry (PIV). This analysis uncovered phase transitions dependent on cell density and alignment, providing physical insights into swarm patterning and behavior. Toward the end, the swarms coalesced to form scattered microcolonies, which were millimeters to centimeters away from the mother colony. This behavior reveals a mechanism that could be akin to seed dispersal in plants or spore dispersal in fungi, potentially increasing the survival probability of swarming bacterial species under adverse conditions.

## Materials and methods

### Bacterial strains, media and growth conditions

*C. ochracea* (ATCC 27872) was cultured on Trypticase Soy (Becton Dickinson) agar plates supplemented with 3 g/L yeast extract and 5 μg/ml Hemin (TSY). The plates used in this study had varying concentrations of Bacto agar (Becton Dickinson), between 0.5% and 2%. To investigate how blood changes swarming, 5% defibrinated horse blood was added to a subset of the plates (TSY-blood). *C. ochracea* was maintained on TSY with 1.5% agar, in an anaerobic chamber at 37°C with a final gas mixture of 80% N_2_/17.5% CO_2_/2.5% H_2_. For swarm plate assays, petri plates were incubated in anaerobic boxes (Becton Dickinson) with a high CO_2_-producing anaerobic sachet (Mitsubishi) and four small flasks containing ~30 ml H_2_O each to maintain a high relative humidity. *C. ochracea* Δ*gldK* strain, a gift from Kazuyuki Ishihara from Tokyo Dental College, was used as a control for motility [18].

### Swarm assays on varying surface stiffness and nutrient composition

**Figure 1:**
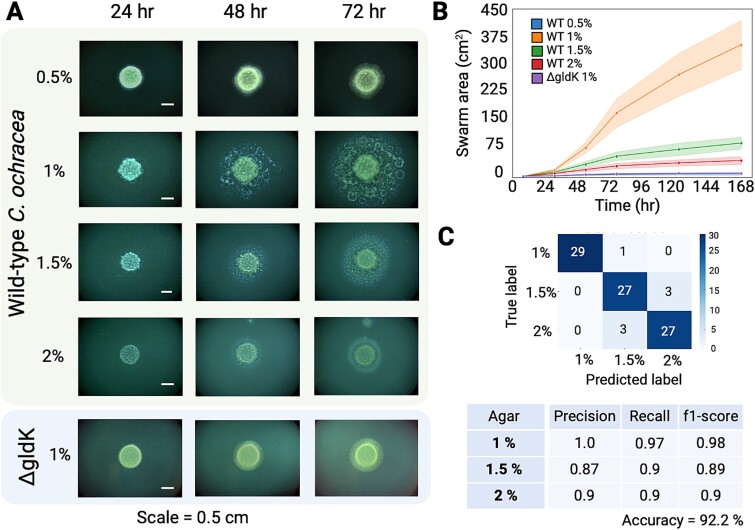
***C. ochracea* swarms exhibit distinct phenotypes on surfaces of varying stiffness.** Cell suspensions spotted on TSY agar plates were incubated anaerobically for 7 days. Images were taken at 24, 48, 72, 96 and 168 h. **(A)** Images of wild-type swarms demonstrate that different swarm patterns and sizes result from varying surface stiffness. The T9SS mutant *ΔgldK* is used as a motility-deficient control. **(B)** Swarm areas throughout the course of development indicate that swarms on 1% agar are significantly larger than swarms on the other agar concentrations. **(C)** A CNN trained on a subset of swarm images from TSY plates with 1%, 1.5%, and 2% agar achieved an accuracy of ~92% in classifying test images. The associated precision, recall, and F1-scores highlight the reproducibility of the swarm patterns. The color bar represents the number of images evaluated by the trained algorithm.

Swarm assays were performed on TSY plates with and without blood, and with 0.5%, 1%, 1.5% or 2% agar. Plates were made fresh on day 0 and allowed to set for 30 min before inoculation. A 2-day old *C. ochracea* swarm plate was scraped in 1 ml TSY broth, and the OD_600_ was adjusted to 0.8. A volume of 5 μl of the culture was spotted on fresh plates and air dried in a laminar flow biosafety cabinet for 30 min. Plates were then transferred to an anaerobe box containing water flasks and incubated under high CO_2_ for 7 days. Swarm size was measured using swarms inoculated on TSY plates, and images were acquired using a stereoscope (AmScope, MU1809) on days 1, 2, 3, 5, and 7. Four biological replicates were acquired. To improve the contrast of images for the Fig. 1, 0.05% black food coloring (Chefmaster liqua-gel coal black food color, Fullerton, CA) was added to the agar (dyed TSY). A preliminary experiment was performed using regular TSY and dyed TSY plates to ensure the dye had no effect on *C. ochracea* swarming (Fig. S1).

### AI-based evaluation of swarm patterns on different agar concentrations

To evaluate the similarity of bacterial motility patterns within three experimental conditions (1% agar, 1.5% agar, and 2% agar), convolutional neural networks (CNNs) were implemented in Python using TensorFlow and Keras. A dataset of 180 images was divided equally into training and testing sets (90 images each) with a 50:50 train-test split. A CNN model, consisting of three convolutional layers (with 32, 64, and 128 filters), max-pooling layers, a dense layer with 128 neurons, and a softmax output layer, was used to classify images. Activation functions, including ReLU, Swish, and GELU, were implemented to determine if similar images within each category could be accurately grouped. The three activation functions provided similar results. Models were trained over 20 epochs using the Adam optimizer and sparse categorical cross-entropy loss and evaluated using metrics such as accuracy, precision, and F1-score.

### Swarm timelapse acquisition

*C. ochracea* swarm development was investigated using a stereoscope over the course of 5 days. TSY with 1% and 1.5% agar were used for swarm tracking. Plates were inoculated as described above, then transferred to a humidified anaerobe box fixed to a stereoscope using double-sided tape, and housed in a 37°C incubator. Timelapses were started at 20 hours to allow sufficient time for the condensation produced by the anaerobe sachets to dissipate so that clear images could be obtained. Images were acquired every 5 minute for ~3–4 days, until the swarm front exceeded the field of view. To ease the burden of image capture and analysis, pixels were binned (4X) to produce images of 1228 × 920 pixels. Timelapses were repeated in triplicate.

### Particle image velocimetry tracking of developing swarms

Movies were first preprocessed in ImageJ [43] to even out the non-constant illumination profile and to reduce noise. For this, a background image obtained from averaging 50 images of virgin agar plates was subtracted from all images in the movie. Next, the [I_min_; I_max_] integer grayscale of the background subtracted images was recast on a 8-bit gray scale ([0; 255]). Finally, a filter was applied to improve signal over noise ratio (S/N). A 3D median filter (σ_X_ = 0 px, σ_Y_ = 0 px, σ_t_ = 3 frames) was applied to the 1% agar movies, but we had to apply a stronger 3D mean filter (σ_X_ = 2 px, σ_Y_ = 2 px, σ_t_ = 3 frames) to the 1.5% agar movies, because the stronger light scattering at this concentration resulted in lower signal:noise.

A previously developed Particle Image Velocimetry algorithm was then applied to the movie to measure local velocities, using its implementation as a publicly available Plugin for ImageJ [44]. The algorithm is explained in depth elsewhere [44, 45]. In essence, it measures the spatial drift Δ***r***(***x***,*t*) of the local light intensity pattern around the interrogation point at position ***x*** from time point *t* to the next frame *t +* Δ*t*. Since this local light intensity pattern is the image of the portion of the bacterial colony in the vicinity of position ***x***, the speed **v** = Δ***r/***Δ*t* corresponds to the average speed of this local group of cells (Fig. S2).

We set the adjustable parameters of the PIV software for our experimental conditions as follows. The spacing of the quadratic grid of interrogation points was *dl* = 8 pixels for 1% agar movies and *dl* = 4 pixels to resolve the smaller spatial features in the 1.5% agar case. The square interrogation window around each point, from which the drift is computed, had a size *l* = 64 pixels, which we kept relatively large to ensure good accuracy. The independently adjustable radius of the spatial pattern taken in consideration for drift calculation was *fl* = 7 pixels for 1% agar movies and *fl* = 10 pixels for 1.5% agar [44]. This choice trades off between underestimating the drift if *fl* is too small and creating spurious spatial correlations of the velocity if *fl* is too large. The algorithm calculates Δ***r*** via a fit of the phase shift of the Fourier components of the spatial pattern, considered up to a maximal wave number *q_max_* [44]. Because the speed of the pattern can be relatively high (up to ~0.5 pixels/frame), we had to reduce the parameter *q_max_* from its default value (3.14 pixels^−1^) down to *q_max_* = *14 ×* 2*π/l* = 1.37 pixels^−1^, to avoid so-called phase wrapping that causes speed under-evaluation (see reference 44, 45 for details). Finally, we set the software to compute the local velocity ***v**(**x**,t)*, which is shown in the figures and used for further analysis, by smoothing Δ***r***(***x***,*t*)***/***Δ*t* on a *tVel* = 10 frames wide sliding window to further reduce noise. Namely, ***v**(**x**,t)* is computed as the slope of the cumulative drift ***r***(***x***,*t*)=${\sum}_{t^{\prime }=0}^t\Delta \boldsymbol{r}\left(\boldsymbol{x},{t}^{{\prime}}\right)$ as a function of time by a linear fit on this sliding window [44] and (Fig. S2).

### Data analysis of PIV timelapses

Custom MATLAB scripts [44, 46] were used to extract velocity maps from the raw PIV data. We then calculated the norm of the velocity, the vorticity and the divergence of microswarmers velocity field using custom Python codes. We computed normalized versions of the vorticity and divergence to dampen the effects of swarmer density being inhomogeneous and focus on gradients of motion directions,$\nabla \times \boldsymbol{v}=\left|\boldsymbol{v}\right|\left(\frac{\partial{u}_y}{\partial x}-\frac{\partial{u}_x}{\partial y}\right)$ and $\nabla \cdotp \boldsymbol{v}=\left|\boldsymbol{v}\right|\left(\frac{\partial{u}_x}{\partial x}+\frac{\partial{u}_y}{\partial y}\right)$, with **u** = **v**/|**v**|. These data were used to characterize temporal and spatial arrangement of *C. ochracea* swarms. Various statistical analyses were performed to quantify swarm development. All data and custom scripts are freely available online on our GITHUB page (https://github.com/amandazdimal/CapnoPIV.git).

### Agent based modeling of swarm behavior

This simulation uses principles of randomization, local interaction (density and alignment), and periodic division to simulate the movement and behavior of cells on a surface. It visualizes how cells interact, align, and move over time, showcasing emergent patterns and behaviors in simulated cellular systems. Individual cells are initially simulated and each cell is initialized with attributes for its position and orientation. Cells are positioned using Cartesian coordinates, offering flexibility in their placement. The initial position $\left({x}_i,{y}_i\right)$ of each cell is determined by a randomly generated radius${r}_{seed}$ which is determined by $R$ multiplied by the square root of a random number between 0 and 1. Here, $R$ is the radius parameter ensuring uniform distribution within a circle. When generating individual cells around a seed point (eg., due to cell-divison), $R$ corresponds to a value of 2. A total of 400 cells are generated per seed and only one seed is used in this simulation. The initial orientation angle of the cell is ${\theta}_i$, and the speed of cellular motion is $v$.


(1) \begin{equation*} {r}_{seed}=R.\sqrt{random\left(0,1\right)} \text{ and } {\theta}_i= random\left(0,2\pi \right) \end{equation*}

(2) \begin{equation*} \left({x}_i,{y}_i\right)=\Big({r}_{seed}.\cos{\theta}_i, {r}_{seed}.\sin{\theta}_i\Big) \end{equation*}

The progression of the simulation is visualized by representing each cell as an oriented rod, which iterates through a series of discrete time steps of 0.1. To reproduce the initial vorticity observed in our experiments an initial $\omega$ (rate of change in orientation) of 0.1 is provided in the simulation. The position of the cell changes over time to simulate the motility of the cell as the following:


(3) \begin{equation*} \left({x}_{new},{y}_{new}\right)=\left({x}_i+v.\cos{\theta}_i. dt,\kern0.5em {y}_i+v.\sin{\theta}_i. dt\right) \end{equation*}

(4) \begin{equation*} {\theta}_{new}={\theta}_i+\omega . dt \end{equation*}

The speed and alignment of each cell are governed by their local environment, with the local density $D\left(x,y\right)$ around a cell calculated as follows:


(5) \begin{equation*} D\left(x,y\right)={\sum}_{i=1}^N\left[\sqrt{{\left({cell}_i.x-x\right)}^2+{\left({cell}_i.y-y\right)}^2}<{R}_{local}\right] \end{equation*}

Here ${R}_{local}$ represents the distance around each cell within which other cells are considered. This defines the scope of the local neighborhood for each cell, and which neighboring cells influence one another. In other words, this determines how far out from a given cell the simulation looks to find neighboring cells. The alignment of a cell is governed by the density threshold, ${D}_t$ which varies from 10 to 50. and the alignment strength, ${A}_{str}$ which varies from 0.07 to 0.21. ${D}_t$ dictates the cell speed based on local density as follows:


(6) \begin{equation*} {v}_a=\left\{\begin{array}{@{}ll}{S}_r,& if\ D\left(x,y\right)>{D}_t\\{}S,& if\ D\left(x,y\right)\le{D}_t\end{array}\right. \end{equation*}

Here, ${v}_a$ is the updated speed of the cell. $S$ is the speed of the cell, and ${S}_r$ is the reduced speed of 4 after the cell encounters a density exceeding ${D}_t$. ${A}_{str}$ determines the degree of alignment with neighboring cells. It influences the orientation of the cell, ${\theta}_{new}$. The average angle (${\theta}_{avg}$) is calculated by first averaging the sine and cosine components of neighboring cells, then finally determined by using the Python function arctan2. ${\theta}_i$ represent the current orientation of the ${i}^{th}$ cell, $n$ is the number of cells within ${R}_{local}$ and $\Delta \theta$ is the shortest angular distance between ${\theta}_{avg}$ and ${\theta}_i$.


(7) \begin{equation*} {\theta}_{avg}=\mathit{\arctan}2\left(\frac{\sum \mathit{\sin}{\theta}_i}{n},\frac{\sum \mathit{\cos}{\theta}_i}{n}\right) \end{equation*}

(8) \begin{equation*} \Delta \theta ={\theta}_{avg}-{\theta}_i \end{equation*}

(9) \begin{equation*} {\theta}_{new}=\left({\theta}_i+{A}_{str}.\Delta \theta \right)\ \mathit{\operatorname{mod}}\ 2\pi \end{equation*}

## Results

### *C. ochracea* swarm patterns and sizes change as a function of nutrient composition and surface stiffness

The area and structure of *C. ochracea* swarms were examined on TSY plates with and without blood, and with adjustments to surface stiffness by altering the agar concentration from 0.5% to 2%. On surfaces of optimal stiffness, *C. ochracea* expanded from the edge of the inoculation spot and this expansion ultimately led to a subset of cells becoming immotile and forming microcolonies. The remaining motile cells continued to swirl outward, thus creating distinct motile and nonmotile regions within the swarm.

Swarming was absent on all plates with 0.5% agar, irrespective of the nutrient composition. The colony sizes on these plates were similar to those of the T9SS deficient, non-motile *ΔgldK* strain (Fig. 1A, S3). Optimal swarming, marked by extensive surface coverage of *C. ochracea* swarms, and a distinctive phenotype characterized by circular and wave-like strings of microcolonies, was observed on plain TSY plates containing 1% agar. Swarms on 1.5% and 2% agar showed patterns similar to one another, although those on 2% agar were smaller and featured tighter, less spread-out microcolonies. On both 1.5% and 2% agar, the microcolonies exhibited more pinpoint or colony-like formations, unlike those observed on plain TSY with 1% agar (Fig. 1A). Additionally, significant differences in swarm sizes on TSY agar with 1%, 1.5%, and 2% agar were observed (Fig. 1B, Table S1). Under laboratory conditions, *Capnocytophaga* species and other human oral microbiome isolates are typically cultured in TSY medium, with or without added blood. TSY blood plates containing 1% agar showed a marked reduction in swarm area, nearly 65% smaller than plain TSY with 1% agar, and exhibited significantly fewer microcolonies. Interestingly, on 1.5% and 2% agar, swarm sizes and patterns remained consistent regardless of blood addition (Fig. 1A, B, S4). Given that the differences in swarm patterns across agar concentrations are observed on both TSY blood (Fig. S4) and TSY-only plates (Fig. 1), we focus on patterns observed on TSY-only plates for the remainder of this study.

To assess the reproducibility of swarm patterns at different agar concentrations, CNNs were trained using TensorFlow and Keras. The CNNs achieved test accuracies of around 92%, indicating that the models reliably distinguished between the data categories. Importantly, the classification performance within each category was consistent, suggesting that most images in a given category (e.g. 1% agar) were similar to one another. The 1% agar category showed near-perfect classification with minimal confusion across other categories, while minor misclassifications occurred between 1.5% agar and 2% agar, possibly reflecting subtle overlaps in image features between these conditions. These overlaps likely reflect minor variances in bacterial swarming that are subtle but biologically plausible. Precision, recall, and F1-scores were used to evaluate the performance of the model. Precision is the proportion of predicted positives that are correct, Recall is the proportion of actual positives correctly identified, and the F1-score is the harmonic mean of Precision and Recall. For 1% agar, Precision was 1.00, Recall was 0.97, and F1 was 0.98. The 1.5% category yielded a Precision of 0.87, Recall of 0.90, and F1 of 0.89, while the 2% category achieved a Precision and Recall of 0.90, and an F1 of 0.90. Overall, these metrics indicate robust classification accuracy, with occasional misclassifications attributable to biological noise. These findings validate that the experimental categories are internally consistent and distinct from other categories (Fig. 1C).

### Tracking spatiotemporal changes in swarm behavior

**Figure 2:**
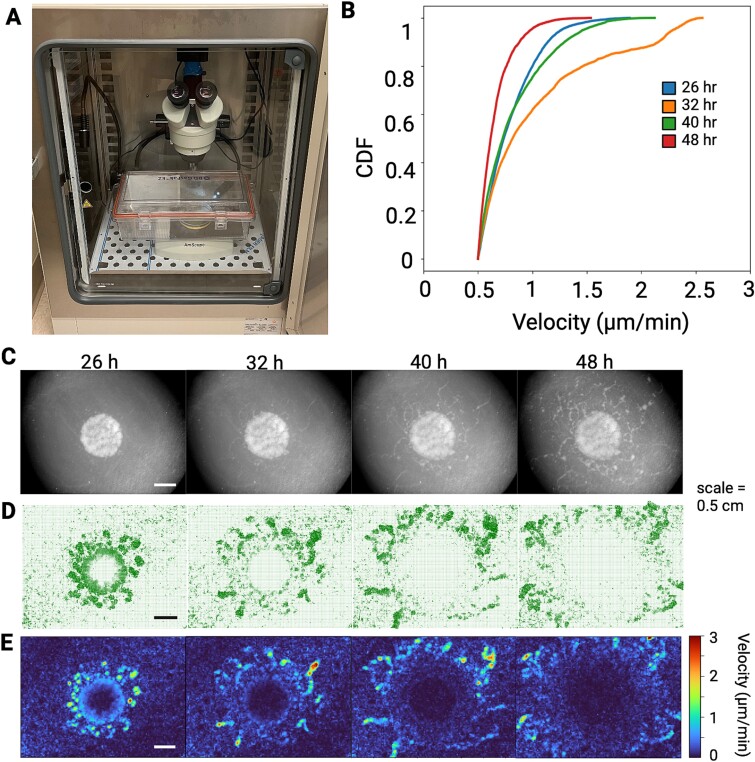
**Developing swarms undergo phase transitions.** Live imaging of developing swarms were captured over the course of two days. **(A)** Live imaging setup for anaerobic swarms. **(B)** A cumulative density function of swarm speeds during the different phases, showing greatest speeds at 32 hours, and slowest speeds at 48 hours. **(C)** Stereoscope images of developing swarms from different timepoints. **(D)** Particle image velocimetry of the swarms allowed tracking of swarm behavior and identification of distinct spatiotemporal features as demonstrated by quiver plots. **(E)** Heatmaps of the velocity of developing swarms measured by image velocimetry. Movies S1 to S12 present biological replicates of swarming bacteria, showcasing the analyses described above.

A live imaging setup (Fig. 2A) was designed, allowing for the capture of time-lapse images of anaerobic swarms of *C. ochracea* over several days (Fig. 2B). Approximately 20 hours after inoculation on agar, the structural features of the swarm start to emerge. Therefore, recordings commenced around 20 hours and continued until the swarm front extended beyond the field of view. Images of swarms on varying agar concentrations were captured every 5 min (Figs. 2, S5 and S6, Movies S1–S4, **Supplementary text).** The swarm behavior of *C. ochrachea* was quantified using a modified version of traditional PIV, which is based on Phase Differential Microscopy (Movies S4–S6). This approach (see methods) calculates the local velocity field $v(r)$ by averaging over several neighboring bacteria. Changes in pixel intensity are employed to assess the speed and directionality of swarm development [44]. Following PIV, velocity maps were extracted and plotted as heatmaps (Movies S7–S9) that demonstrate spatial changes in swarm velocity. Additionally, quiver plots (Movies S10–S12), which display the velocity vectors identified via PIV show the directionality and relative speed of movement of the swarms.

**Figure 3:**
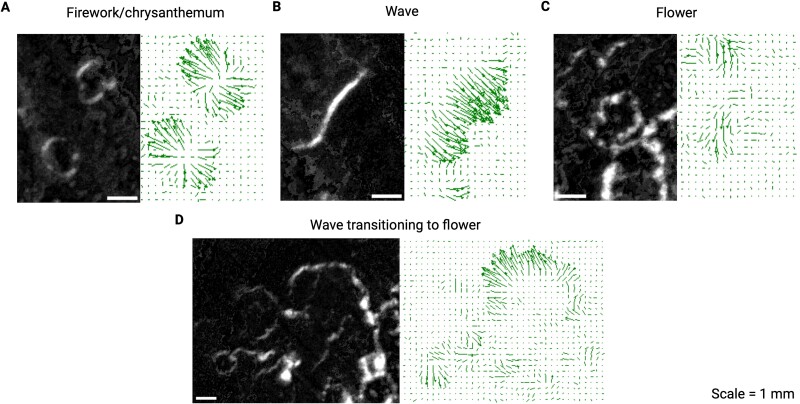
**Microswarm expansion patterns.** Developing *C. ochracea* swarms exhibit different phases of expansion. The zoomed-in swarm image is accompanied by quiver/velocity plot of that region. **(A)** Firework or chrysanthemum-like patterns where swarmers expand in all directions from a central point. **(B)** Wave-like movements where swarmers align along a single plane and move in the same direction causing a swarm front. **(C)** Flower-like movements where swarmers move in broken or irregular circular patterns causing a rippled-like expansion similar to flower petals. **(D)** Transition from wave to flower-like expansion, characterized by breaks in the plane of movement, changes to the directionality of some microswarmers, and the emergence of flower-like structures. A 100-pixel vector represents a velocity of 2 μm/s.

**Figure 4:**
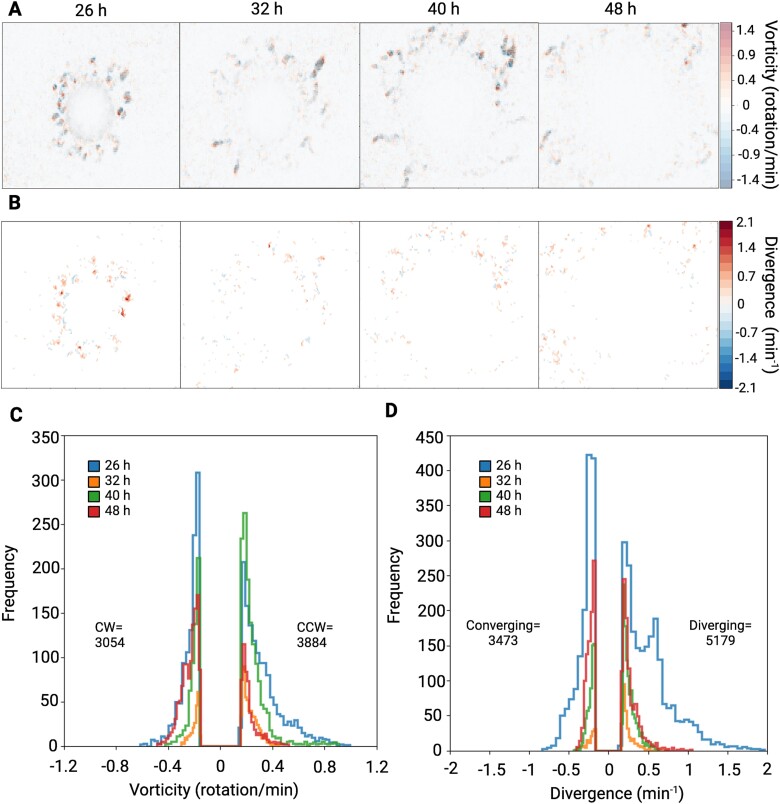
**Cell flow derivatives during swarm development. (A)** Vorticity computed from velocity maps is superimposed on a grayscale velocity heatmap, where positive vorticity values represent counterclockwise rotation and negative values indicate clockwise rotation of swarms. The plots highlight higher vorticity during the initial phase, with reduced rotational movement in later phases. **(B)** Divergence extracted from the velocity map, which quantifies swarmers motion toward or away from a given point in a flow field, with positive values showing divergent movements and negative values indicating convergent movements. **(A, B)** hotspots of high vorticity and divergence correspond to fireworks and flower-like expansion patterns. **(C)** Frequency distribution of vorticity demonstrating clockwise and counterclockwise rotation, showing slight bias for microswarmers to move in a counterclockwise manner. **(D)** Frequency distribution of divergence, showing greater bias for divergent patterns of microswarmers within the fluid flow.

Plotting $v(r)$ of *C. ochracea* swarms on a 2D image plane provided a spatiotemporal map of swarming. This mapping revealed characteristics that distinguish *C. ochracea* swarms from bacteria that employ other motility mechanisms, such as flagellar-driven swarming and the distinct type of gliding motility of Myxobacteria [47, 48]*.* Approximately 26 hours after inoculation, *C. ochracea* swarms were primarily active around the periphery of the initial inoculation area. Small groups of swarming cells, hereafter referred to as micro-swarmers, emerged from the inoculation region. Interestingly, the micro-swarmers initially appeared as irregularly shaped offshoots disconnected from the parent colony (Fig. 2C, 2E). However, many micro-swarmers expanded radially, displaying a pattern similar to fireworks bursting with a chrysanthemum effect (Fig. 3A). This pattern of movement by the microswarmers was characterized by high flow vorticity, i.e. counterclockwise rotational movement, and large divergence, quantifying the expansion from the center of the chrysantemum (Fig. 4A–C). These bursting micro-swarmers later transitioned to a flower-like pattern, characterized by circular forms with jagged edges (Fig. 2D, Fig. 3C, D, Movie S7). The Cumulative Distribution Function (CDF) displays the arrangement of the $v(r)$ measured throughout the timelapse. At 26 hours $v(r)$shows a range from 0.5 μm/minute to 1.5 μm/min, characterized by a long tail, indicating the presence of a subset of faster moving cells within the majority of slower cells.

Around 32 hours, in the areas with the fastest moving swarms, the flower-like patterns divided into two jagged semi-circular shapes. Typically, the semi-circle closer to the periphery expanded outwards, displaying a wave-like pattern of high velocity and low vorticity (Fig. 3B, Fig. 4A). As a wave of swarming bacteria expanded, it merged with another asynchronous wave, forming long and thin wave-like patterns with differing periodicity (Movie S1). The CDF shows the range of $v(r)$ expands from 0.5 μm/minute to over 2.5 μm/min, suggesting an increase in $v(r)$ and possibly in cellular metabolism as well. Notably, a pronounced peak in the CDF between 2 μm/minute and 2.5 μm/minute at 32 hours suggests that a distinct subset of the cellular population has differentiated itself from the rest of the slower-moving swarm and the faster moving cells exhibit the wave-like motion described above (Fig. 2E).

By 40 hours, the central region near the initial inoculation spot significantly slowed down, with most motility observed in the periphery. Some of the waves reverted to a more radial-like expansion, with a slight increase in vorticity. Additionally, the swarm velocity at 40 hours resembles the swarm velocity measured at the initial 26 hours (Fig. 2B).

Around 48 hours and onwards, the speed of motility substantially decreased, and large dendritic patterns of bacterial growth were observed. This was accompanied by a drop in overall vorticity (Fig. 4A). Due to the different types of expansion patterns reported here, temporal changes in vorticity were observed, with the maximum vorticity occurring at ~1.5 rotations per minute. (Figs. 4A and C, S7 and S8). Our data indicate that swarms vortex in both clockwise and counterclockwise directions. While majority of swarms show bias for counterclockwise vortexing, much like swarms of bacteria that utilize flagella for motility [27, 49, 50]. In one case no bias was observed throughout the duration of the experiments, although the signal to noise ratio was higher in this experiment (Figs. 4, S7, S8, Movies S10–S12). We also found that *C. ochracea* microswarmers commonly exhibit divergent behavior as compared to convergent behavior (Figs. 4D & S7), but in one case, due to the high signal to noise ratio, similar convergent and divergent activities were observed (Fig. S8).

Towards the end of the timelapse, the majority of $v(r)$ measurements were less than 1 μm/minute and scattered microcolonies of *C. ochracea* are observed (Fig. 2B). In fact, most of the swarms were now non-motile, and due to the swarm patterns described earlier, the overall density of bacteria on the agar plate was unevenly distributed. Interestingly, the regions with higher bacterial concentrations later developed into scattered micro-colonies that were several millimeters to centimeters away and completely disconnected from the parent colony (Figs. 2C-E). These temporally-driven changes in swarm speed were supported in the additional timelapses generated via this study (Figs. S5 & S6). Overall, our analysis demonstrate two major modes of movement for *C. ochracea* swarms, one in a wave like pattern where the cells line up and create a linear front, and the second being more radial or fireworks-like expansion where the cells appear to expand outward in all directions while rotating from a central area. This speed and type of movement was not found on 1.5% agar (Supplemental text, Fig. S9), indicating a specificity of this type of development on softer surfaces.

### Developmental phase transitions are observed within swarms

The data described above led us to a hypothesis that swarming *C. ochracea* might have distinct developmental phases. In the imaging data, these phase transitions manifest as spatiotemporal shifts in swarm patterns, including wave-like expansions and radial (fireworks-like) bursts, corresponding to different motility regimes. We observe that during the initial phase, ~20–26 hours after inoculation, micro-swarmers emerge at the periphery, displaying localized bursts of motility, indicative of a transition from a low-motility state to a more dynamic phase. As the swarm matures between 26 and 40 hours, faster-moving cells form flower-like, fireworks-like, and wave-like patterns, suggesting a collective transition to a more organized, high-energy state, characterized by increased velocity. By 40–48 hours, a significant reduction in motility is observed, along with the formation of scattered microcolonies, indicating a transition back to a lower-energy state as the swarm reorganizes into static clusters. These temporal phase transitions reflect an adaptive balance between motility, environmental conditions, and collective bacterial interactions. To test for the presence of phase-transitions, we plotted the magnitude of $v(r)$ as a function of the swarm density corresponding to each $v(r)$ value, and found that three distinct phases emerged that correspond to swarm propagation and pattern formation at different time points. The significantly motile regions, where the velocity exceeded 0.5 μm/minute, had pixel intensity ranging from 0.3 to 0.7 (Fig. 4A & S10). At 26 hours, the peak speed was 1.5 μm/minute. At 32 hours, it increased to 2.5 μm/minute and at 40 hours, it dropped back to 1.5 μm/minute. By 48 hours, the speed fell below 1 μm/minute and the pixel intensity ranged from near zero to 0.7.

Additionally, all timepoints displayed an overlap in the 0.5–0.7 μm/minute regime, indicative of slow-moving groups that form microcolonies. This behaviour appears to contribute to the floral patterns and isolated swarmers described above for *C. ochracea* swarms. In a contrasting hypothetical scenario, if all cells only slowed around 48 hours to form microcolonies, one might observe a ring-like pattern typical of *E. coli* swarms [47]. Hence, the distinct phases, with some overlap, suggest a novel developmental strategy that allows for random placement of the microcolony away from the parent colony, potentially increasing the survival probability of the species.

**Figure 5:**
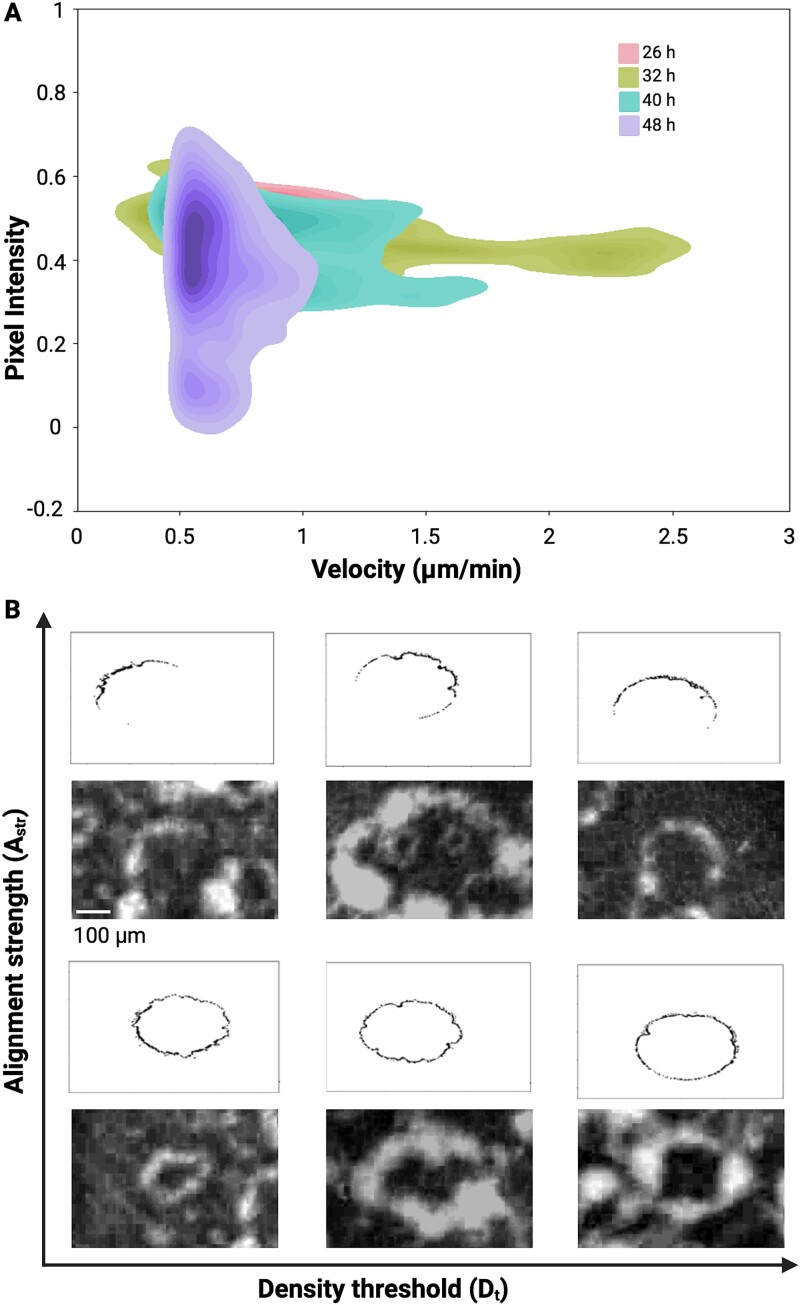
**Swarm development model.** An agent-based model was developed to characterize microcolony swarm expansion. **(A)** Kernel density (KDE) plots demonstrating different phases during swarm development via changes in velocity and pixel intensity (i.e. cell density). Pixel intensity values between 0.3 and 0.5 demonstrate the highest speeds at all time points, with the maximum speed achieved at 32 hours. **(B)** Swarm simulations under low and high alignment strengths and density thresholds, and representative images of microcolonies with similar properties to those created in the simulation. A representative simulation is displayed in Movie S13. Swarms with low alignment strength create connected chains of microcolonies, generating more circular phenotypes than those with high alignment. Swarms with high density threshold create more smooth swarm fronts as compared to lower densities.

As *C. ochracea* swarms grow, microcolonies scatter across the agar plate, resulting in uneven cell density—higher in some regions and lower in others. Similarly, in certain microcolonies, the rod-shaped cells align parallel to each other, while in other areas, individual cells are oriented more orthogonally. To better understand the physical forces that drive the spatial structuring and energetics of the *C. ochracea* swarm, we developed a numerical agent-based model to simulate the cellular dynamics and spatial patterning observed in our experimental data. This model, which focuses on cell movement, orientation, and collective behavior, reveals that both alignment and local density play a crucial role in shaping the spatiotemporal structure of *C. ochracea* swarms (Fig. 5A). The numerical model represents cells as individual entities, each possessing spatial and angular properties in a 2D space. The entities are first positioned within a circle using polar coordinates, with R representing the radius of the circle. As time progresses the positions of the cells transform into Cartesian coordinates within the 2D space. The progression of the simulation is visualized by representing each cell as an oriented rod, which iterates through a series of discrete time steps. The position of the cell changes over time to simulate the motility of the cell.

This model encompasses the position updates for cells in Cartesian coordinates, based on their initial position, velocity, and orientation, with adjustments for local cell density influences on speed and alignment.


(1) \begin{align*} & \left({x}_{new},{y}_{new},{\theta}_{new}\right) \nonumber \\ &\qquad =\left({x}_i+{v}_a.\mathit{\cos}{\theta}_i. dt,{y}_i+{v}_a.\mathit{\sin}{\theta}_i. dt,{\theta}_i+\omega . dt+{A}_{str}.\Delta \theta \right) \end{align*}

(2) \begin{equation*} {v}_a=\left\{\begin{array}{@{}ll} Sr & if\ D\left(x,y\right)>{D}_t\\{}S & if\ D\left(x,y\right)\le{D}_t\end{array}\right. \end{equation*}

Here, ${v}_a$ represents the speed of the cell, ${\theta}_i$is the initial orientation angle, $\omega$ is the rate of change of orientation. ${A}_{str}$is the alignment strength, and $\Delta \theta$ is the angular adjustment based on local alignment. The term $dt$ represents the discrete time step. $S$ is the standard speed while $Sr$ is the reduced speed when the local density exceeds${D}_t$. This numerical model effectively captures the dynamics of cell movement and interaction as they migrate outward from the inoculation spot on a simulated surface. The velocity transition from $S$ to $Sr$ is currently defined as a step function and future iterations of the model will explore smoother transitions to potentially identify thresholds where behavioral shifts become pronounced. The results from our model demonstrate the emergence of swarm patterns observed in the timelapses (Fig. 5B). For example, in several field of views from one of the timelapses we can detect nearly all formations that were generated by the simulation. Increasing ${A}_{str}$ results in less radial or more wavelike patterns, whereas increasing ${D}_t$ generates swarms with less breaks or protrusions in the swarm pattern, causing more smooth edges. In situations with high ${A}_{str}$ and low ${D}_t$, non-uniform (i.e. flower petal-like) wavelike pattens are created (Fig. 5B, Movie S13). High${A}_{str}$ and moderate ${D}_t$ form incomplete and non-uniform radially expanding swarms. Low ${A}_{str}$ and high ${D}_t$ results in complete radial expansion without many breaks or indentations in the uniform (i.e. continuous curvature) circular swarm. Lastly, low ${A}_{str}$ and low ${D}_t$ results in mostly complete and non-uniform radial expansion with indentations but not many gaps in the swarm front (Fig. 5B). While our empirical data shows essentially all modes of swarming predicted by the simulation, in the experimental conditions, we typically observe swarms with all ${A}_{str}$ values and low to moderate ${D}_t$ values. We don’t typically observe radially expanding swarms without any breaks or protrusions in the swarm, as predicted in models with high ${D}_t$. Hence, we find that the different phases that lead to spatiotemporal patterning of *C. ochracea* swarms depend on changes in cell-density, alignment strength and motile speed.

## Discussion

Motility is a survival strategy used by organisms across all scales of life. Tracking of multicellular organisms has demonstrated motility as a means of survival and relocation to areas of more optimal living conditions [51]. Similarly, fungi release spores, and plants perform seed dispersal to ensure species survival and colonization of new locations [52, 53]. This activity is synonymous with bacteria using motility as a means to inhabit new locations. Interestingly, we find that the structure demonstrated by *C. ochracea* swarms produce dispersed microcolonies which potentially increases the survival probability of the species.

Bacteria have developed diverse motility strategies to relocate and scavenge for nutrients, demonstrating parallels in evolution. The implications of vastly different species evolving machineries that enable their motility demonstrates the importance of active movement in evolutionary fitness. The diverse types of bacterial motility lead to differing expansion strategies and swarm patterns. In a co-culture, *Acinetobacter baylyi* and *E. coli* have been shown to create flower-like patterns [24] and monocultures of *E. coli* are capable of generating chiral flows that are faster than the swarming cells [25]. *Myxococcus xanthus* has been shown to create 3D fruiting body structures under low nutrient conditions [32]. *Bacillus subtilis* forms branched, concentric rings [54] that are developed by microscopic whirls and jets [26]. *Serratia marcescens* and *Flavobacterium johnsoniae* display vortices that control their resulting swarm phenotypes [27, 55].

Tracking and modeling of swarm characteristics of a common anaerobic oral microbe *C. ochracea* provides novel insight into survival strategies used by bacteria. The formation of small, pioneering propagation fronts of *C. ochracea*, which we observe strikingly when the “fireworks” first shoot off, is generally absent in swarms of well-studied bacteria, underscoring the uniqueness of this system. We find that phase transitions underlie the swarm patterns of coordinated *C. ochracea* cells. These transitions are governed by cell density and alignment strength. The cell density can be increased either through population growth, or through organization of cells via cell surface adhesins. For example, interaction between SprB adhesins on the surfaces of neighboring cells could increase local cell density within micro-swarming populations. Similarly, contact between SprB proteins could create higher alignment strength between cells, causing them to move in a coordinated fashion from physical adherence between cells. External factors could also influence alignment strength of cells, including fluid flows, or neighboring micro-swarmers.

We report that different speed regimes correlate with the types of spatial expansion patterns exhibited by the swarm. Wave-like movements of cells appear to achieve higher speeds than radial or flower-like swarm patterns. This may indicate two different strategies that underlie swarming. When collectively moving cells exhibit high alignment strength and move along the same axis, they may be able to quickly escape an area. Conversely, utilizing a more radial-type expansion at slower speeds results in cells covering larger surface area, and may demonstrate a scavenging or nutrient seeking phenotype. These observations also align with changes in vorticity over time and we find that swarms of gliding bacteria frequently diverge from the parent swarm. Taken together, these might indicate that swarms of T9SS-driven gliding bacteria display specialized motility tactics in a temporally driven fashion as development proceeds.

We have identified surface stiffness, cell-density, alignment strength, and motile speed as factors that influence spatiotemporal patterning of *C. ochracea* swarms. Future investigation on how metabolism, respiration, chemotaxis, cell division, and polymer production influence spatiotemporal patterning [56–61] might lead to the identification of controllable knobs that can be tweaked to alter the spatial structure of *C. ochracea* swarms. Our current model permits multiple cells to occupy the same spatial coordinates and captures the fundamental features of the observed swarm patterns. While this approach allowed us to test the significance of alignment strength and cell-density, future efforts could incorporate collision mechanics which may prove valuable in scenarios where nutrient or chemoattractant gradients are tested experimentally, potentially providing deeper insights into how jamming or packing might shape the spatial structure of gliding *C. ochracea* swarms. In the human oral microbiota, bacteria from the genus *Capnocytophaga* are found in abundance in supra-gingival and sub-gingival biofilms [19]. In *in-vitro* settings, non-motile microbes and phages often hitchhike on gliding *Capnocytophaga*, consequently altering the structure of polymicrobial communities. The data presented here show that swarms of *C. ochracea* exhibit spatial patterning, which could potentially serve as a template for seeding the spatial structure of the oral microbiota in a polymicrobial context. Building on this, future studies could investigate how phase transition within bacterial swarms influence resource sharing, competition, and bet-hedging within microbial communities. Future research exploring the effects of swarming on the spatiotemporal patterning of swarms will not only enhance our understanding of the swarm behavior of gliding *C. ochracea* but will also shed light on its potential role in shaping the structure of oral polymicrobial communities.

## Supplementary Material

Supplementary_material

Movie_S1_wrae263

Movie_S2_wrae263

Movie_S3_wrae263

Movie_S4_wrae263

Movie_S5_wrae263

Movie_S6_wrae263

Movie_S7_wrae263

Movie_S8_wrae263

Movie_S9_wrae263

Movie_S10_wrae263

Movie_S11_wrae263

Movie_S12_wrae263

Movie_S13_wrae263

## Data Availability

Custom Python codes for image analysis and example datasets are freely available on our GitHub https://github.com/amandazdimal/CapnoSwarmAnalysis. The software for PIV analysis is available on https://doi.org/10.5281/zenodo.3516258 and GitHub https://github.com/croelmiyn/FourierImageAnalysis. All other results and data are included in the article, and/or supplemental material.
