# Supplementary material for: Swarming bacteria exhibit developmental phase transitions to establish scattered colonies in new regions
Source: ISME J. 2025 Jan 3;19(1):wrae263. doi: 10.1093/ismejo/wrae263 (PMC11773418; doi:10.1093/ismejo/wrae263)
Supplement: Supplementary_material [file supplementary_material.pdf]

Supplementary Information

Swarming bacteria exhibit developmental phase transitions to establish scattered colonies in new regions

Authors. Amanda M. Zdimal[1, 2], Giacomo Di Dio[3], Wanxiang Liu[1, 2], Tanya Aftab[1], Taryn Collins[1], Remy Colin[3], and Abhishek Shrivastava[1, 2]

Affiliation. [1]Center for Fundamental and Applied Microbiomics, Biodesign Institue, Arizona State University, Tempe, AZ 85287, [2]School of Life Sciences, Arizona State University, Tempe, AZ 85287, [3]Department of Systems and Synthetic Microbiology, Max Planck Institute for Terrestrial Microbiology, Marburg, Germany 35043

SUPPLEMENTARY FIGURES

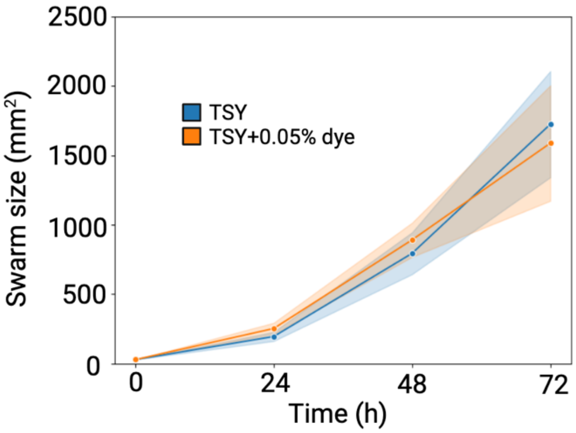

Figure S1. Addition of 0.05% dye on TSY plates with 1% agar show no effect on swarm size. *C. ochracea* swarming was tested on plain TSY with 1% agar, and TSY with 1% agar and 0.05% black food coloring to examine swarming changes in the presence of the dye. There were no statistical differences between conditions for any of the timepoints tested. Data represents 4 biological replicates.

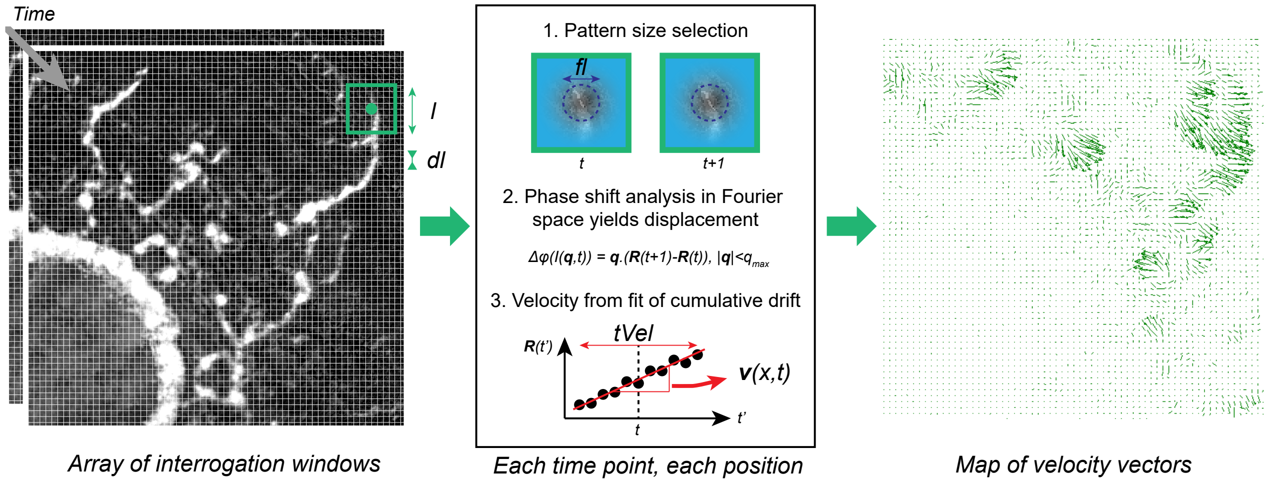

Figure S2. A schematic of the image velocimetry method.

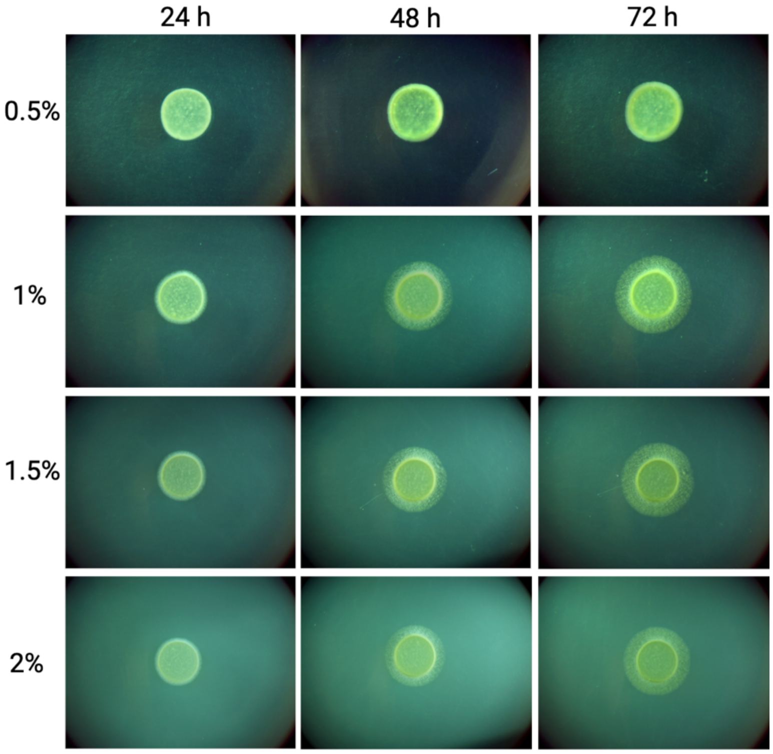

Figure S3. Swarming deficient *ΔgldK* on TSY plates with varying agar concentration. Plates were prepared and inoculated as described in **Figure 1**, but spotted with *C. ochracea* ΔgldK cell suspensions. Swarming was not observed, although colony expansion was evident as cells divided and spread out over time.

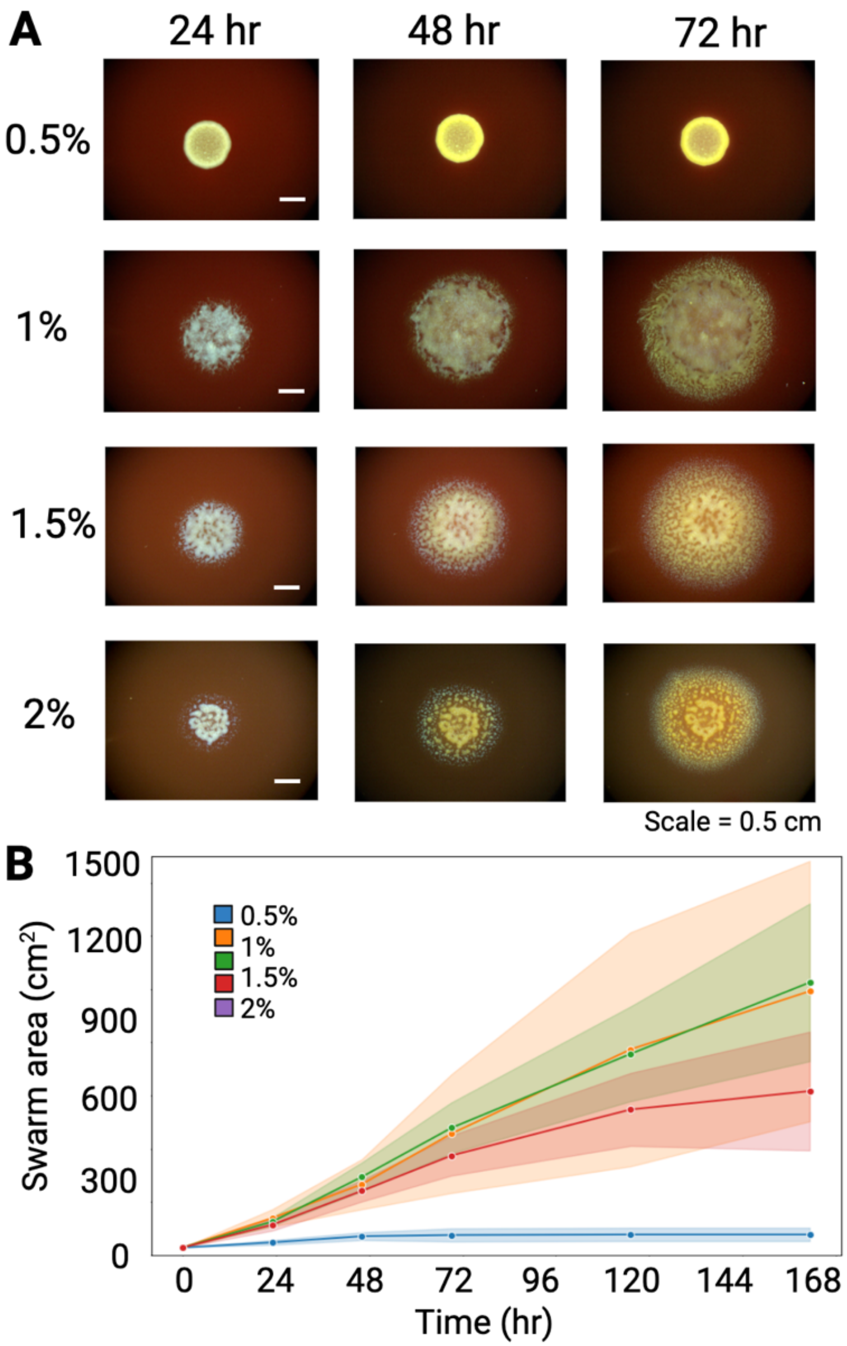

Figure S4. Swarm development on TSY-blood plates. Swarms were tracked as described in **Figure 1**, however these plates contained 5% defibrinated horse blood. **(A)** Photos of *C. ochracea* swarms on TSY-blood plates of varying surface stiffness. **(B)** Swarm area was tracked throughout the duration of the experiment, which showed no statistical significance between 1% and 1.5% agar, unlike results on TSY plates.

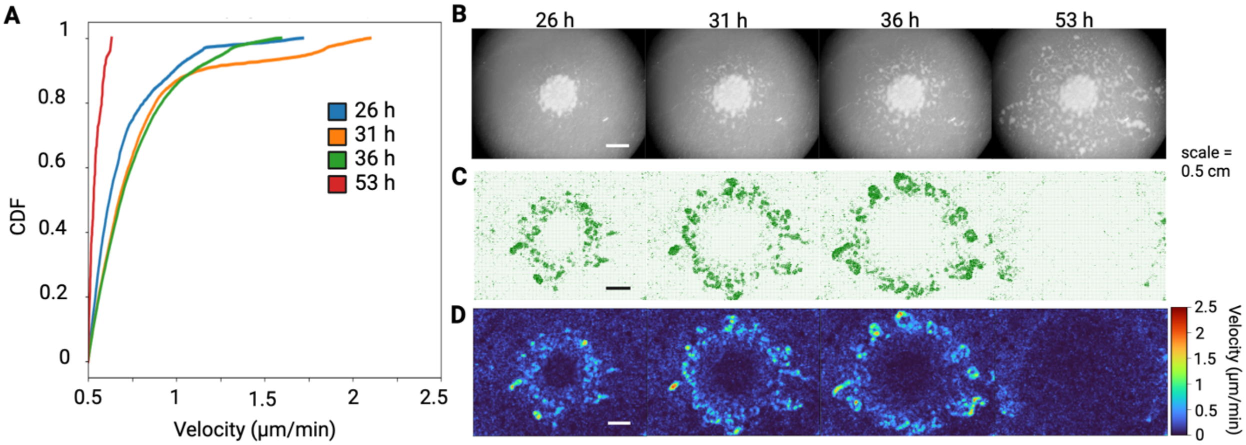

Figure S5. Phase transitions during swarming in timelapse 2. Live imaging of developing swarms were captured as described in **Figure 2**. **(A)** Cumulative density function (CDF) plots for swarm speeds. **(B)** Stereoscope images, **(C)** quiver plots and **(D)** heatmaps of developing swarms during the 4 phases.

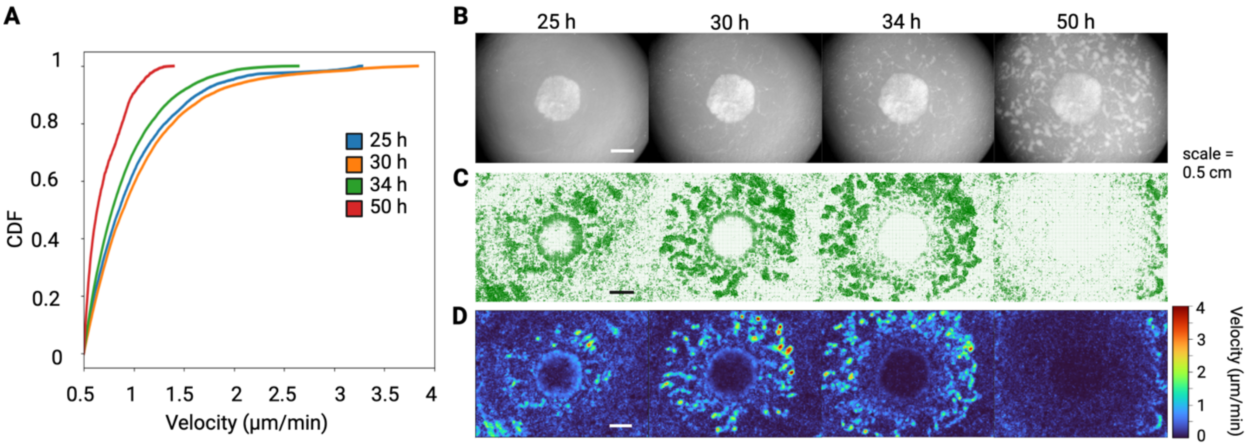

Figure S6. Phase transitions during swarming in timelapse 3. Live imaging of developing swarms were captured as described in **Figure 2**. This timelapse had a different signal to noise profile at the edges as compared with timelapse 1 and timelapse 2. **(A)** Cumulative density function (CDF) plots for swarm speeds. **(B)** Stereoscope images, **(C)** quiver plots and **(D)** heatmaps of developing swarms during the 4 phases.

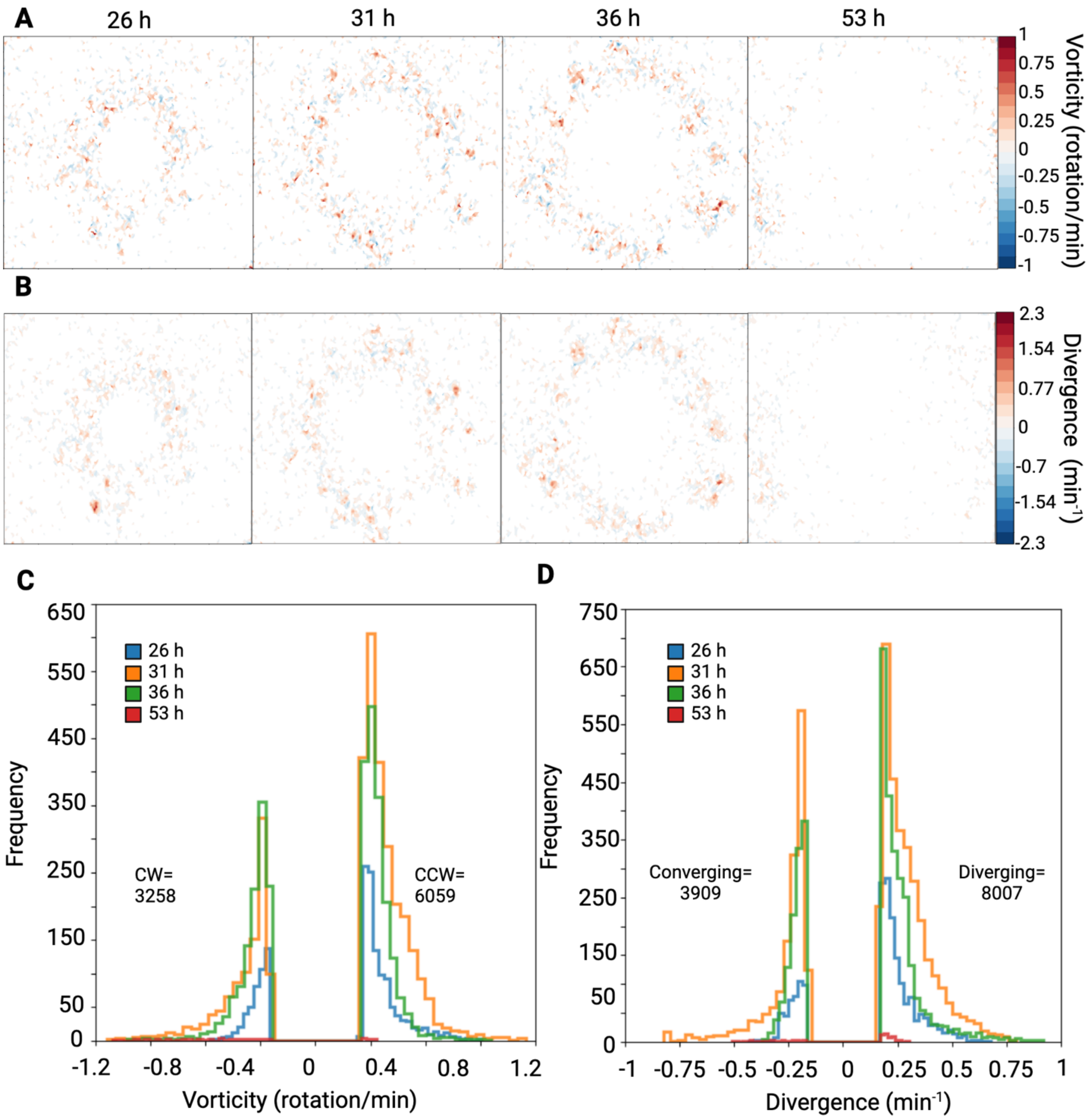

Figure S7. Cell flow derivatives during swarm development, timelapse 2. Velocity maps were used to generate vorticity and divergence data as described in **Figure 4**. **(A)** Vorticity plots showing very similar high vorticity patterns at 31 h and 36 h in phases 2 and 3, with lower rotational movements in early and late phases. **(B)** Divergence plots showing either converging or diverging movements within the flow **(C)** Frequency distribution showing clockwise and counterclockwise rotation, with nearly double the amount of CCW movements vs CW. **(D)** Frequency distribution of divergence, demonstrating significantly more microswarming diverging activity within the flow.

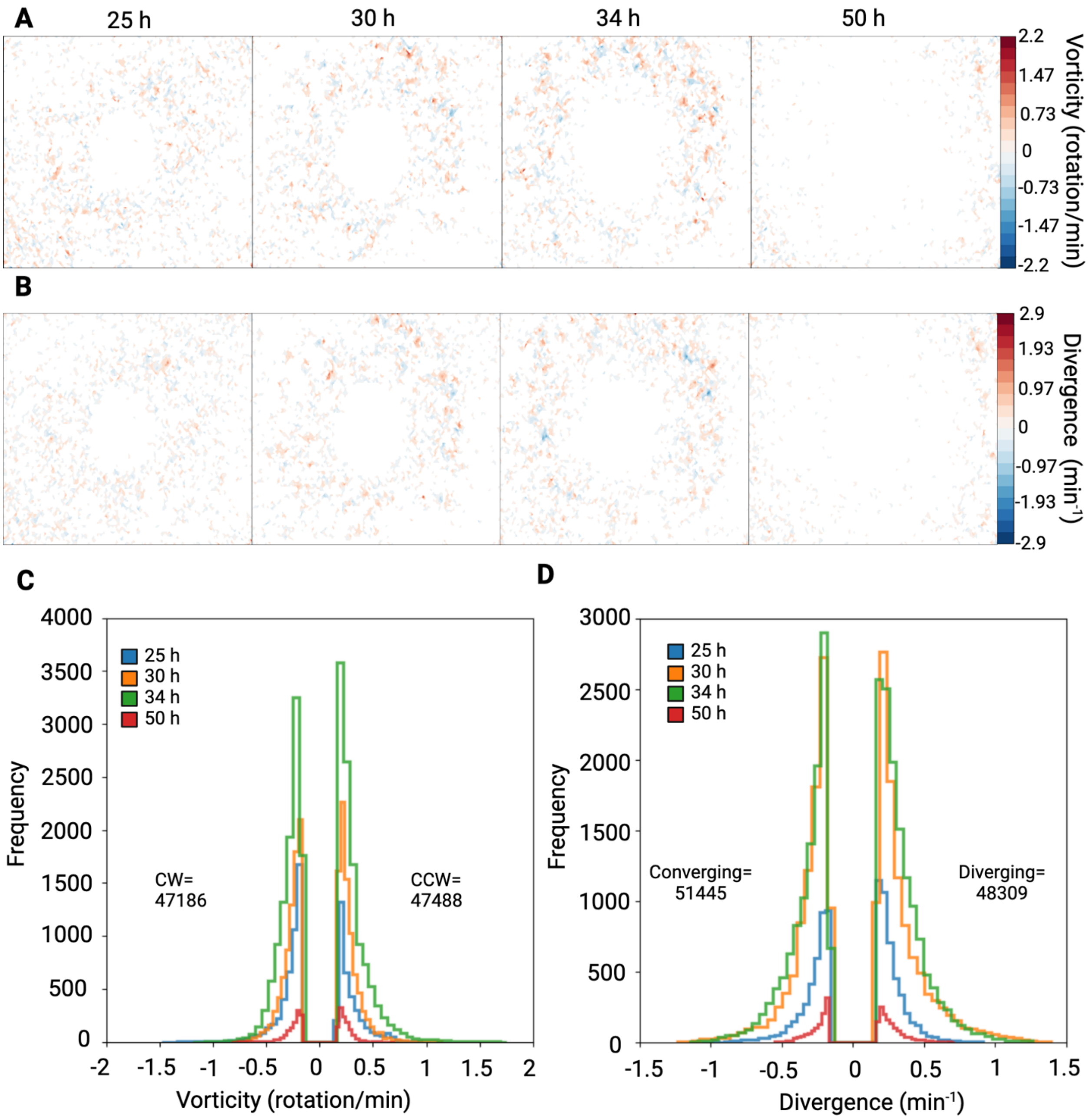

Figure S8. Cell flow derivatives during swarm development, timelapse 3. Velocity maps were used to generate vorticity data as described in **Figure 4**. **(A)** Vorticity plots showing highest vorticity at 30 h and 34 h in phases 2 and 3, and lowest rotational movements in early and late phases, much like **Figure S7**. **(B)** Divergence plots showing a similar amount of converging or diverging movements within the flow, unlike **Figures 4 and S7**. **(C)** Frequency distribution of vorticity demonstrating relatively similar clockwise and counterclockwise rotations. **(D)** Frequency distribution of divergence, demonstrating a slight bias for converging movements, which is in contrast to the majority of diverging activity in timelapses 1 and 2.

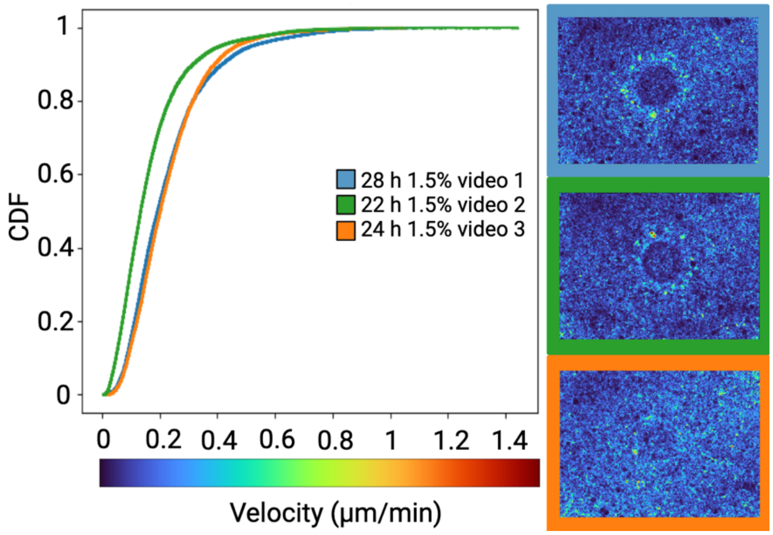

Figure S9. Microswarmer speed on 1.5% agar. Timelapses of developing swarms were captured on 1.5% agar in the same manner for 1% agar. $v(r)$ was determined for timelapse videos in triplicate. The frames demonstrating the fastest speeds for each timelapse was chosen for CDF analysis. The corresponding heatmaps for the frames are displayed on the right of the plot. Velocities remained below 1.5 μm/min in all 3 videos, demonstrating much slower swarm development on 1.5% agar.

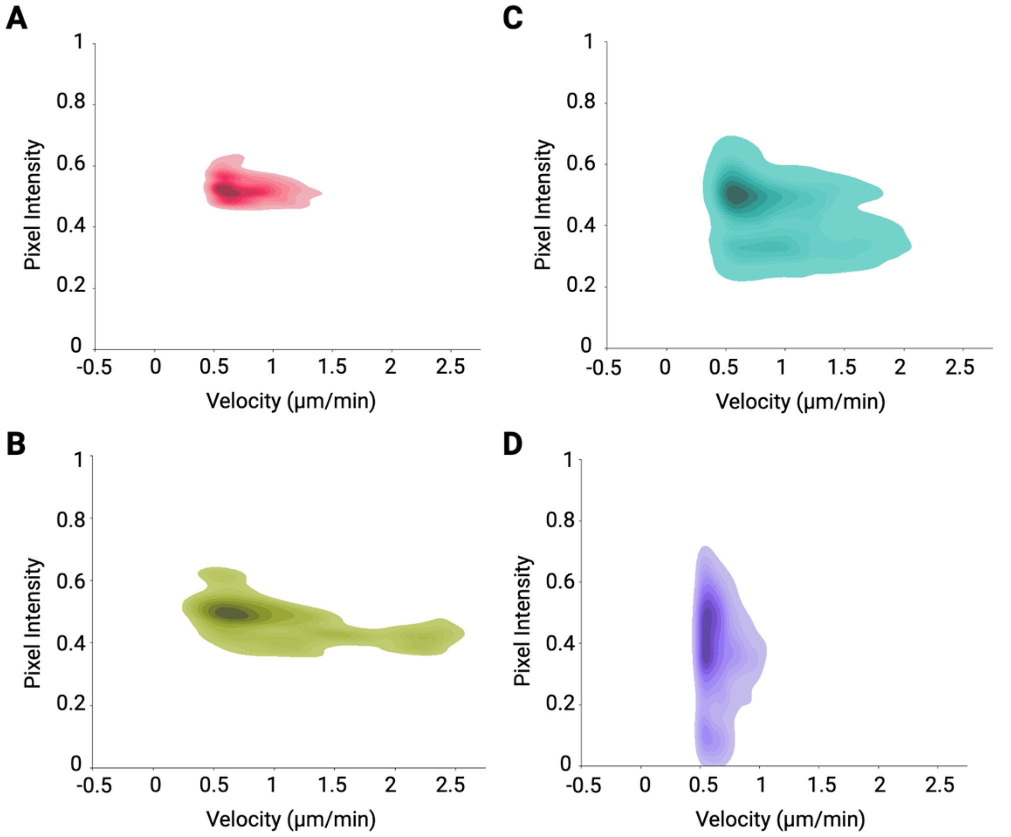

Figure S10. Phase transitions during swarm development. Kernel density (KDE) plots were generated as described in **Figure 4A**. These are the individual KDE plots for the swarm at 26 h **(A)**, 32 h **(B)**, 40 h **(C)**, and 48 hr **(D)**.

Table S1. Statistical comparison of *C. ochracea* swarm sizes on TSY agar. An ANOVA was performed to investigate statistical significance of swarm size at each individual time point. Significance was seen with all comparisons at each time point. (*p ≤ 0.05, ** p ≤ 0.01, *** p ≤ 0.001, **** p ≤ 0.0001).

Comparison	0 h	24 h	48 h	72 h	120 h	168 h
**0.5% vs 1%**	n.s.	****	****	****	****	****
**0.5% vs 1.5%**	n.s.	****	****	****	****	****
**0.5% vs 2%**	n.s.	****	****	****	****	****
**1% vs 1.5%**	n.s.	**	****	****	****	****
**1% vs 2%**	n.s.	****	****	****	****	****
**1.5% vs 2%**	n.s.	*	***	***	****	****

SUPPLEMETARY MOVIES

Movie S1. Timelapse (example 1) of the developing swarm on 1% agar described in Figure 2. The movie records swarm development from 24 h to 55 h.

Movie S2. Example 2 of a developing swarm on 1% agar. The movie records swarm development from 24 h to 54 h.

Movie S3. Example 3 of a developing swarm on 1% agar. The movie records swarm development from 24 h to 48 h.

Movie S4. Particle image velocimetry of the swarm shown in movie S1. Orange arrows depict the velocity vectors superimposed on the raw images.

Movie S5. Particle image velocimetry of the swarm shown in movie S2. Orange arrows depict the velocity vectors superimposed on the raw images.

Movie S6. Particle image velocimetry of the swarm in movie S3. Orange arrows depict the velocity vectors superimposed on the raw images.

Movie S7. A velocity heatmap of the swarm in movie S1 shows spatial segregation of the swarm velocity.

Movie S8. A velocity heatmap of the swarm in movie S2 shows spatial segregation of the swarm velocity.

Movie S9. A velocity heatmap of the swarm in movie S2 shows spatial segregation of the swarm velocity.

Movie S10. Quiver plots depict the velocity vectors of the swarm shown in movie S1.

Movie S11. Quiver plots depict the velocity vectors of the swarm shown in movie S2.

Movie S12. Quiver plots depict the velocity vectors of the swarm shown in movie S3.

Movie S13. Timelapse of a numerical simulation that explains the spatial features observed in the developing swarms of gliding bacteria.

SUPPLEMENTARY TEXT

Macroscopic swarm development was also investigated on TSY with 1.5% agar and 2% agar. However, due to imaging limitations, 2% swarms were omitted from this report due to poor resolution from imaging through the denser agar in these plates. Swarm development on 1.5% agar was tracked using the same method as was described using the 1% agar plates. Similar to the limitation on 2% agar plates, the denser agar and slower movement of swarms made PIV tracking more difficult. However, we were able to generate swarm speed from the data collected from PIV. The swarms on 1.5% agar tend to move slowly, maxing out at speeds around 1.5 μm/min (**Figure S9**), which is 3-times slower than the 4.5 μm/min max speed seen in swarms on 1% agar.
